# Effect of Liming with Various Water Regimes on Both Immobilization of Cadmium and Improvement of Bacterial Communities in Contaminated Paddy: A Field Experiment

**DOI:** 10.3390/ijerph16030498

**Published:** 2019-02-11

**Authors:** Lei Shi, Zhaohui Guo, Fang Liang, Xiyuan Xiao, Chi Peng, Peng Zeng, Wenli Feng, Hongzhen Ran

**Affiliations:** Institute of Environmental Engineering, School of Metallurgy and Environment, Central South University, Changsha 410083, China; shilei1121@126.com (L.S.); fliang@issas.ac.cn (F.L.); chipeng@csu.edu.cn (C.P.); zengzengpp@foxmail.com (P.Z.); fwlandy@csu.edu.cn (W.F.); qxlrql@csu.edu.cn (H.R.)

**Keywords:** lime, microbial community, potentially toxic elements, soil remediation, agronomic measures

## Abstract

Cadmium (Cd) in paddy soil is one of the most harmful potentially toxic elements threatening human health. In order to study the effect of lime combined with intermittent and flooding conditions on the soil pH, Cd availability and its accumulation in tissues at the tillering, filling and maturity stages of rice, as well as enzyme activity and the microbial community in contaminated soil, a field experiment was conducted. The results showed that liming under flooding conditions is a more suitable strategy for in situ remediation of Cd-contaminated paddy soil than intermittent conditions. The availability of Cd in soils was closely related to the duration of flooding. Liming was an effective way at reducing available Cd in flooding soil because it promotes the transformation of Cd in soil from acid-extractable to reducible fraction or residual fraction during the reproductive growth period of rice. Compared with control, after liming, the concentration of Cd in brown rice was reduced by 34.9% under intermittent condition while reduced by 55.8% under flooding condition. Meanwhile, phosphatase, urease, and invertase activities in soil increased by 116.7%, 61.4% and 28.8%, and 41.3%, 46.5% and 20.8%, respectively. The high urease activity in tested soils could be used to assess soil recovery with liming for the remediation of contaminated soil. Soil microbial diversity was determined by the activities of soil acid phosphatase, urease and available Cd by redundancy analysis (RDA). The results indicated that the problem of Cd-contaminated paddy soil could achieve risk control of agricultural planting by chemical treatment such as lime, combined with various water regimes.

## 1. Introduction

Contamination of paddy soils with potentially toxic elements has become a worldwide environmental problem with the extensive development of industrial activities [1,2,3], over-fertilization [4], and irrigation with water contaminated with toxic elements [5,6]. They can accumulate in the edible parts of crops, enter the human body through the food chain and thus threaten human health [7,8,9,10,11]. Rice (*Oryza sativa* L.) is one of the world’s most important crops and is consumed daily in Asia. In the south of China, the pH value of the soil is generally acidic. The rice grown in paddy soils with low levels of Cd-contamination can easily exceed the Chinese food safety standard of 0.20 mg/kg (GB 2762–2017). Rice is considered as a major source for Cadmium (Cd) in the diet of humans [12,13,14], so soil Cd contamination has led to public concern and it is imperative to develop suitable technologies to guide planting of rice in lightly and moderately Cd-contaminated paddy soils.

Recently, remediation technologies for potential toxic element contamination in soil have been developed, such as chemical immobilization [15,16,17], water management [18,19,20], chemical washing [21,22], and bioremediation [23,24,25]. In situ chemical immobilization is an especially effective method to control the availability of potentially toxic elements [26,27].

Amendments, such as lime, organic amendments, and clay minerals and so on, are added to soil to reduce the availability of potentially toxic elements. Lime is one of the most cost-effective and widely used amendments to reduce potentially toxic element uptake by plants. The number of studies has been published regarding the use of lime alone or in combination with other inorganic additives applied as soil amendments for the remediation of toxic element-contaminated soil in pot experiments [28,29,30,31]. Liming at 1250–1500 kg/ha could reduce the rice grain Cd content by 35.3% in a Cd-contaminated paddy field [32] while at 1200 kg/ha in rice field during the tillering stage it can decrease the content of Cd in rice grain by 15% [33]. Besides, cycling between wetting and flooding states during the growth of rice could also affect the accumulation of Cd in rice [18,31,34]. However, the effect of lime combined with different water regimes on the immobilization of Cd in contaminated paddy fields has rarely been reported.

The evaluation for remediation of potentially toxic element-contaminated soil includes the availability of potentially toxic elements, the impact of plant-based bioassays and restoration of soil function [35,36]. Crop yield and accumulation of potentially toxic elements are used to evaluate remediation of potentially toxic elements-contaminated soil. Soil microbes and soil enzyme activities play an important role in the sustainable development of soil systems, and are considered the most sensitive potential indicators to characterize the changes of soil quality [36,37]. Soil enzyme activity and microbial community diversity have often been proposed as important indicators following the restoration of soil function [37,38]. Reducing cadmium toxicity in cadmium-contaminated soil would improve the soil microbial community structure [35,37]. Meanwhile, soil enzymes are closely related to soil microorganisms, they are secreted by microorganisms, and participate in material circulation and energy flow together with the microorganisms. There is a certain correlation between soil enzyme activity and the degree of available Cd contamination. Previous studies have reported soil urease, invertase, acid phosphatase and dehydrogenase are sensitive to Cd contamination, which could reflect the toxic effects of Cd [35,38]. Many studies on paddy soil remediation have focused on reducing the Cd uptake by rice, but few have considered the effect of lime combined with water regimes on soil enzymes and microorganisms. Therefore, the objectives of this study were: (1) to study the effects of liming on soil Cd availability and the accumulation of Cd in rice using field cultivation on Cd-contaminated paddy soil under intermittent and flooding conditions and, (2) to assess the influences of liming on soil enzymatic activity and the microbial community under intermittent and flooding conditions.

## 2. Materials and Methods

### 2.1. Characterization of Tested Soil and Amendment

The tested paddy field is located in Xiangtan County (Hunan Province, China, 27°49′38.10″ N and 112°51′30.70″ E). The county has a subtropical monsoonal climate, with a mean annual temperature of 16.5 °C, mean annual sunshine duration of 1670 h, and a mean annual rainfall of 1350 mm. The basic properties of the soil were as follows: pH, 5.01 ± 0.32; organic matter, 35.02 ± 3.24 g/kg; available nitrogen, 85.34 ± 5.26 mg/kg; available phosphorus, 3.04 ± 0.84 mg/kg; available potassium, 35.68 ± 2.52 mg/kg; total Cd, 1.42 ± 0.31 mg/kg; total Pb, 79.0 ± 4.20 mg/kg; cation exchange capacity, 14.36 ± 2.42 cmol/kg; clay, 61.2%; silt, 17.4%; sand, 21.4%. Lime was purchased from a local market and had a total Cd content of 0.47 ± 0.06 mg/kg.

Soil pH is measured using a 1:2.5 (*w*/*v*) mixture with deionized water without CO_2_. The organic matter content was determined by K_2_Cr_2_O_7_ oxidation. The available N in soil was determined by an alkali hydrolysis and diffusion method. The available P was determined by 0.5 M NaHCO_3_ and analyzed by the molybdenum antimony-ascorbic acid colorimetric method. The available K was extracted by 1.0 M NH_4_OAc and analyzed by flame photometry [39]. The available Cd in soil was extracted by diethylenetriaminepentaacetic acid (DTPA) [40,41]. The chemical speciation of Cd in soil was determined by sequential extraction procedure (European Community Bureau of Reference) [42]. Plant and soil samples were digested according to the reference [35]. The concentration of Cd in digested solutions was determined using an Inductively Coupled Plasma Emission Spectrometer (ICP-MS, Agilent 7500 Series, Waltham, MA, USA). A quality control analysis was performed with certified reference material of China (GSS-5) and Hunan rice (GSB-23), yielding an analytical error <10%.

### 2.2. Experimental Setup in the Field

The lime dosage was selected according to the reference [43,44]. A total of four treatments were applied in the field with triplicates. The experimental setup is as listed in Table 1.

The square of each plot was 35 m^2^ (5 × 7 m) and was randomly arranged. There was a protective row around the test area to prevent rice cross-pollination between adjacent treatments, and the plot ridges were covered with plastic film to prevent them from collapsing and running water between plots. One week before the transplanting of rice seedlings, a fertilizer consisting of 450 kg/ha mixed fertilizer (N/P_2_O_5_/K_2_O = 1:0.5:1) was applied. Rice seedlings were transplanted with three seedlings per hill at a spacing of 20 cm × 25 cm. Urea was applied for topdressing at the tillering stage of rice. Then the rice cultivar Wufengyou 569 was transplanted into the paddy soil on 20 July 2015. Management measures were conducted according to the traditional production methods.

### 2.3. Sampling and Pretreatment

Five rice plants in each plot were collected and mixed as a sample and three replicate samples were taken from each plot. The corresponding rhizosphere soil was separated by gently shaking it from the roots according to previous reports [45,46]. The specific sampling time is shown in Table 2.

Plant samples were separated into three parts (root, straw, and brown rice) and placed in a 105 °C for 30 min, and then at 60 °C until the weight of the sample remained constant. The dried biomass was ground with a stainless-steel crusher. For rhizosphere soil, one part was air-dried, crushed, and passed through a 2.0 mm and 0.15 mm sieve mesh, while another part was stored at −20 °C prior to a polymerase chain reaction/denaturing gradient gel electrophoresis (PCR/DGGE) analysis. All samples were stored in cleaned polyethylene bags for further analysis.

### 2.4. Soil Enzyme Activity and Microbial Community

Soil urease activity (UA) was assayed with a method described by the reference [47] and expressed as NH_4_-N mg/g/d. Soil acid phosphatase activity (ACP) was determined using the *p*-nitrophenylphosphate colorimetric method [48]. Soil invertase activity (SA) was determined by the method [49] and expressed as glucose mg/g/d. Soil DNA extraction and PCR-DGGE amplification was executed according to previous report [50].

### 2.5. Data Analysis

All data analysis was handled using Excel 2010. Shapiro-Wilk normality tests were used to check the normality of data. Based on the results of normality tests, one-way analysis of variance (ANOVA), a paired sample *t*-test, and non-parametric statistical tests were conducted using SPSS 18.0 (SPSS Inc., Chicago, IL, USA). *p*-values < 0.05 were considered significant. The Shannon diversity index (H) of the genetic diversity of 16S rRNA genes was estimated by the following equation:

$$H=-\overset{s}{\underset{i=1}{\sum }}p_{i}lnp_{i}$$

*H =* where *p_i_* is the ratio of the intensity of a single band to the total intensity of all bands within the same lane, *S* is the total number of bands in each sample lane, and *i* is the order of the total number in each sample lane.

## 3. Results and Discussion

### 3.1. Distribution of Cadmium in Various Parts of Rice

**Figure 1:**
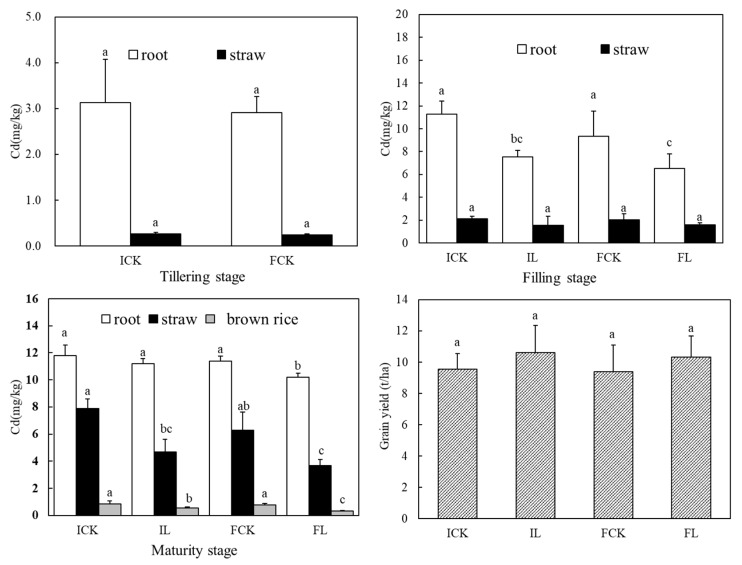
Cd concentration in rice tissues at different growth stages of rice and rice grain yield. ICK, IL, FCK, and FL are the intermittent treatment, lime combined with intermittent treatment, the flooding treatment, and lime combined with flooding treatment, respectively. Data are means ± SD of three replicates. Bars with different letters indicate a significant difference (*p* < 0.05).

The yield of the rice grain is lower under flooding condition than that under intermittent condition. The yield of the rice grain was increased and changed slightly with liming, compared to the control (Figure 1).

The allocation of Cd concentration in rice is shown in Figure 1. Compared to intermittent conditions, the Cd concentration in various parts of rice was decrease slightly under flooding conditions. After liming, the allocation of Cd in rice was significantly different during the different growth stages of rice. At the filling stage of rice, compared to control, the Cd concentration in root was significantly decreased by 33.3% in the intermittent combined with lime treatment (IL), while the corresponding figure was 30.3% in the flooding combined with lime treatment(FL). The Cd concentration of straw was decreased and changed slightly among treatments. At the maturity stage of rice, Cd uptake by rice straw significantly reduced by 40.3% in the IL treatment and 41.7% in the FL treatment, respectively, compared to control. Similarly, compared to control, rice Cd concentration reduced by 34.9% (decreasing from 0.86 to 0.56 mg/kg) in the IL treatment and 55.8% (decreasing from 0.77 to 0.34 mg/kg) in the FL treatment, respectively.

Normally, potentially toxic element concentrations in brown rice are significantly affected by straw and rice root concentrations [51,52,53]. Previous studies have shown that the Cd concentration in rice root and straw was significantly decreased by application of water regimes or amendment [19,54]. With liming, Cd concentration in the root decreased significantly at the filling stages of rice, whereas at maturity stage there was lower Cd accumulation in straw than that in root. The results show that the pregnant stage of rice is the critical periods for controlling rice Cd concentration. Meanwhile, to reduce rice Cd concentration, controlling Cd accumulation in root at the filling stage of rice should be prior considered. In the current study, liming at tillering stage of rice is an effective way to decrease rice Cd concentration under flooding condition.

### 3.2. Soil pH and Availability of Cadmium

Soil pH is an important factor governing solid-solution equilibria of potentially toxic elements [55,56]. As shown in Table 1, the value of pH in flooding soil was higher than that in intermittent soil. Compared to control, soil pH significantly increased (*p* < 0.05) with liming under intermittent and flooding conditions, respectively, because the release of hydroxyl ions through the hydrolysis of lime neutralized the acidity soil. Soil pH was correlated negatively with the availability of soil Cd. The degree of Cd uptake by plants depends on their availability, while DTPA-extractable Cd is suitable for predicting the availability of Cd in soil [24].

Soil DTPA-extractable Cd concentration decreased during growth stages of rice (Table 3). Flooding soil had lower DTPA-extractable Cd concentration than intermittently wetted soil. Compared to intermittent conditions, the DTPA-extractable Cd concentration under flooding treatment was reduced significantly by 24.5% at the filling stage of rice (*p* < 0.05). At filling stage of rice, DTPA-extractable Cd reduced significantly by 18.4% in IL treatment compared to control. At maturity stage of rice, soil DTPA-extractable Cd reduced significantly by 23.0% in IL treatment and 21.6% in FL treatment compared to control, respectively.

Previous studies also stated that flooding conditions generally decreased Cd availability in tested soil [18,19,20,57]. Lime combined with flooding conditions was a more suitable way to reduce soil Cd availability than flooding conditions alone. Several mechanisms have been attributed to the soil Cd availability. First, the increase of pH led to an increase in negative charges of soil under the flooding condition alone or combined with liming and it could also hydrolyze Cd^2+^ to CdOH^+^, which Cd in soil precipitates as hydroxides or carbonates and adsorbs tightly to soil colloid, ultimately, leading to lower availability [34,58,59,60]. Second, the concentrations of iron and manganese oxides in flooding soil have been decreased, while that of mobile Cd in soil increased, which can lead to the immobilization of Cd by readsorption or precipitation [61,62]. Finally, microorganisms in flooded soil, such as sulfur-reducing bacteria that can reduce sulfates to sulfide or S^2−^ which then reacts with Cd^2+^ to form CdS precipitates, can also reduce the availability of Cd [63,64,65].

In addition, after liming, the decrease amplitude of DTPA-extractable Cd concentrations is higher at the maturity stage of rice than that filling stage under flooding conditions. The Cd concentration is low at the maturity stage of rice, indicating that a new equilibrium was established between the different Cd forms in soil, which may be closely associated with soil properties, temperature and rhizosphere environment at different stages of rice. The specific reason needs further research.

**Figure 2:**
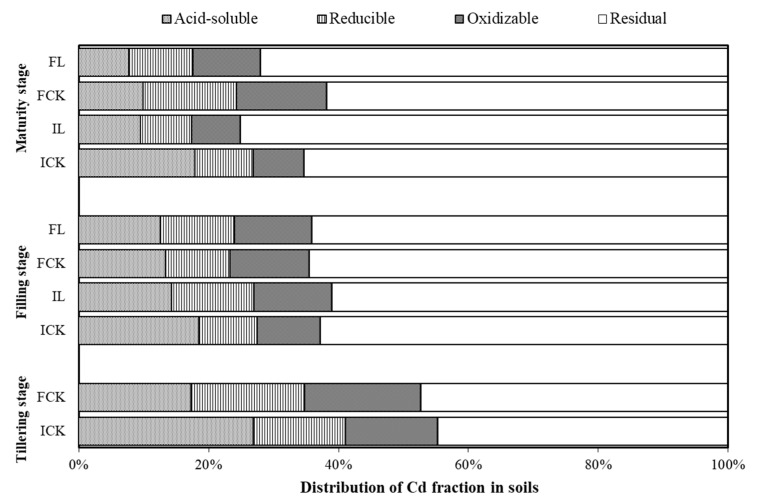
Percentages of Cd fractions in soil at different growth stages of rice. ICK, IL, FCK, and FL are the intermittent treatment, lime combined with intermittent treatment, the flooding treatment, and lime combined with flooding treatment, respectively.

Sequential extraction is often to study the relative bioavailability of soil-sorbed potentially toxic elements by revealing the speciation of the elements in soil [42]. Compared to intermittent wetting, at the maturity stage of rice the proportion of acid extractable Cd decreased significantly by 44.4% in flooded soil while reducible Cd and oxidizable Cd increased by 59.8% and 78.6%, respectively (Figure 2). At the filling stage of rice, the proportions of acid extractable Cd decreased significantly by 22.6% in IL soil and 5.4% in FL soil, while reducible Cd increased by 40.4% and 13.6%, compared to control, respectively. At the maturity stage of rice, the proportions of acid extractable Cd decreased significantly by 47.1% in IL soil and 23.0% in FL soil while residual Cd increased 15.0% and 16.6%, compared to control, respectively. The results indicate that the Cd fractions in soil were closely related to the duration of flooding. Liming promoted the transformation of Cd in soil from acid-extractable to reducible form at the filling stage of rice and to residual fraction at the maturity stage of rice. These results were consistent with Chen, who report that liming can was a suitable way to decrease Cd availability and increased stable fractions under flooding condition [34]. Huang et al. [66] also reported that the combination of moisture management and amendment promoted the transformation of Cd in red paddy soil from acid-extractable to reducible fraction.

### 3.3. Soil Enzyme Activity and Microbial Characteristics

Soil enzyme activity and microbial community have been used to evaluate the soil quality following soil remediation activities [37,38]. Soil urease and invertase activities were reduced by 15.8% and 6.5% under flooding conditions, respectively, compared to intermittent conditions (Table 4). After liming, soil enzyme activity was increased. Phosphatase, urease, and invertase activities in the IL soil were significantly increased by 116.7%, 61.4% and 28.8%, compared to control, respectively. Similarly, in the FL soil, soil urease activity increased by 46.5%, that of acid phosphatase was 41.3%, and that of invertase was 20.8% compared to control, respectively.

Previous studies had also reported that soil enzyme activities were negatively correlated with soil moisture, which was due to the low redox potential and anaerobic soil conditions [67]. The activity of soil enzymes was higher after lime treatment, indicating that a certain degree of metabolic recovery was related to the liming of Cd-contaminated soil. Sun et al. [38] reported that application of sepiolite significantly increased soil enzyme activity and presumed the changes in pH may be primarily responsible for this behavior. However, enzyme activity may also change under potentially toxic element stress [68,69]. In our study, liming changed significantly Cd stress level in the soil, which is another factor that can influence enzyme activity. In addition, the high invertase and urease activities in soils indicated the rich functional state of the soil. Urease activity increased significantly with liming while the invertase changed slightly (Table 4). The results can contribute to the reasons as follows: one the hand, urease activity was significantly affected by the level of contamination due to urease could be combined with soil main component of humus to form stable compounds outside the cells [70,71]. On the other hand, urease is an extracellular enzyme and inhibited by metal ions through reaction with the sulfhydryl groups, synthesis of metal-sequestering saccharides or proteins and trapping or precipitation of metals on microbial surfaces [72,73]. Therefore, urease has the potential to be used to assess soil recovery for the remediation of potentially toxic elements in contaminated soil.

**Figure 3:**
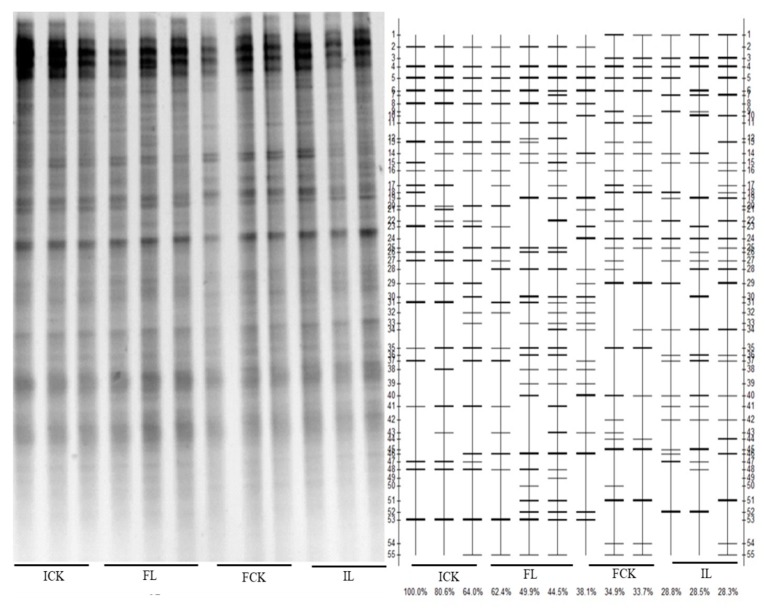
Denaturing gradient gel electrophoresis (DGGE) profile of rhizosphere bacteria communities in contaminated soils.

The composition of the bacterial community plays a role in determining the intrinsic stability of soil microbial communities [74]. DGGE, as a microbial diversity screening method, can monitor the changes of microbial community response at the molecular level [75]. The DGGE band pattern of 16S rDNA amplified by primers 357f-gc and 517R amplification of was used to determine the bacterial community, as shown in Figure 3. The DGGE profiles of bacteria were basically similar after the four treatments, suggesting that the microorganisms with these bands were relatively stable and less affected by the treatments such as liming or water regimes. However, there were still a few bands that emerged or vanished with liming. The changes of bacterial community in soil were presented by the DGGE profiles, and the number of bands in the DGGE patterns increased with liming. In particular, the band number significantly increased with the IL treatment, as shown in Table 2. The Shannon index indicated that bacterial community diversity was slightly reduced in flooding soil, compared to intermittent, while liming significantly increased bacterial community diversity. Soil moisture has consistently been shown to be strongly correlated with the variation in the microbial community [67]. The highest band number and Shannon index can be obtained by application of lime, which could be attributed to the high pH and low toxicity of potentially toxic elements [67,76] or might be relevant for the replication of new bacterial species. A study of the changes of the specific bacteria in soil will be determined by high-throughput sequencing technique. The results indicate that lime combined with various water regimes is favorable to improve soil environmental quality.

### 3.4. Relationships among the Bacterial Community and Environmental Variables

**Figure 4:**
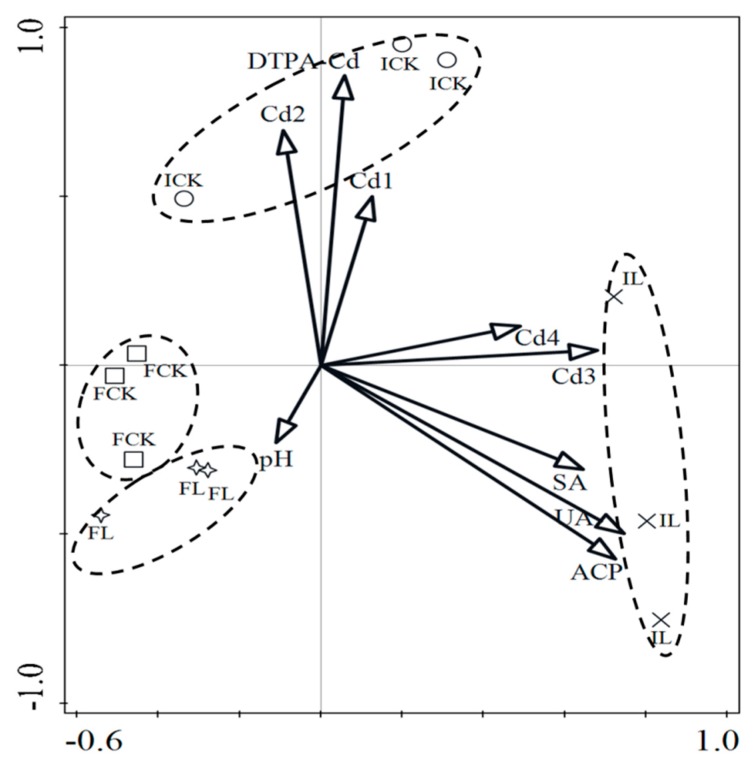
Redundancy analysis (RDA) of the correlation of environmental parameters and bacterial species based on different treatments. DTPA-Cd stands for soil available Cd. Environmental variables were represented as arrows. The length of the arrows manifested the relative importance of that environmental factor in explaining the variation of bacteria and community structures, while the angles between the arrows reflected the degree of the correlations.

Soil microbial community diversity was significantly changed between intermittent and flooding conditions alone or combined with liming (Figure 4). For bacteria between environmental variables and species date, the eigenvalues of the axes 1 and 2 data were 32.5% and 20.4%, respectively. The cumulative percentage of variance represented by the first two axes was 52.9%. The results showed that microbial community was a good indicator for evaluating water regimes treatment alone and combined with liming for the remediation of Cd-contaminated soil and the improving of soil ecological environment quality. Redundancy analysis (RDA) stated that soil microbial community was sensitive to soil available Cd, enzyme activities, liming, and the various water regimes and determined by the activities of soil ACP, UA, SA, the concentration of DTPA-Cd, reducible Cd, and oxidizable Cd according to the length of vectors. Combined with the results on the accumulation of Cd in rice, soil enzyme activities and the beneficial association between microorganisms and liming in soil indicated that lime combined with flooding condition was a suitable way to the remediation of Cd-contaminated paddy soil.

## 4. Conclusions

Liming under intermittent and flooding conditions can significantly reduce soil Cd availability and Cd uptake in rice, and improve the quality of the paddy soil environment. Soil Cd availability was closely related to the duration of flooding. Available Cd concentration in flooded soil was lower than that in intermittently wetted soil and can cause lower Cd concentrations in rice. The reproductive growth period of rice plays an important role in controlling Cd accumulation in straw and roots. Liming at the tillering stage of rice reduced significantly the Cd concentrations in brown rice under flooding conditions while it increased soil urease activity and microbial diversity compared to control. Urease has the potential to be used to assess soil recovery for the remediation of potentially toxic elements in contaminated soil. The soil microbial community determined by the activities of soil phosphatase, urease, invertase and available Cd concentration could be used to effectively evaluate the remediation of Cd-contaminated soil. Therefore, lime combined with flooding condition was adapted for the remediation of Cd-contaminated paddy.

## Figures and Tables

**Table 1:** Experimental setup.

Treatment	Amendment/Management
Intermittent condition (ICK)	Without lime and maintained the depth of surface water at 3.0–5.0 cm until the full tillering stage followed by intermittent irrigation
Intermittent condition + lime (IL)	Liming 1500 kg/ha at the tillering stage of rice and same as the ICK for water management
Flooding condition (FCK)	Without lime and the plot was flooding during the crop growth season and maintained the depth of surface water at 3.0–5.0 cm
Flooding condition + lime (FL)	Liming 1500 kg/ha at the tillering stage of rice and same as the FCK for water management

**Table 2:** Sampling time of each treatment.

Treatment	Rice Growth Stage		
	Tillering Stage (18 August)	Filling Stage (13 October)	Maturity Stage (10 November)
ICK	Sampled	Sampled	Sampled
IL	N	Sampled	Sampled
FCK	Sampled	Sampled	Sampled
FL	N	Sampled	Sampled

Notes: N indicates non-sampling. ICK, IL, FCK, and FL are the intermittent treatment, lime combined with intermittent treatment, the flooding treatment, and lime combined with flooding treatment, respectively.

**Table 3:** Soil pH and available Cd at different growth stages of rice.

Treatment	Tillering Stage		Filling Stage		Maturity Stage	
	pH	Cd	pH	Cd	pH	Cd
ICK	5.65 ± 0.11a	0.41 ± 0.06a	5.73 ± 0.07c	0.34 ± 0.019a	5.75 ± 0.17c	0.36 ± 0.044a
IL	–	–	5.96 ± 0.19b	0.27 ± 0.003b	6.03 ± 0.15b	0.28 ± 0.023bc
FCK	5.63 ± 0.13a	0.37 ± 0.05a	5.81 ± 0.14bc	0.27 ± 0.030b	6.02 ± 0.30b	0.31 ± 0.013ab
FL	–	–	6.16 ± 0.16a	0.25 ± 0.013b	6.38 ± 0.22a	0.25 ± 0.011c

Notes: Data are means ± SD of three replicates. Means followed by the different letter within the same column are significantly different (*p* < 0.05). ICK, IL, FCK, and FL are the intermittent treatment, lime combined with intermittent treatment, the flooding treatment, and lime combined with flooding treatment, respectively.

**Table 4:** Soil enzyme activities and microbial diversity index values for different treatments.

Treatment	Band Number	Shannon Index	Urease (NH_4_-N mg/g)	Acid Phosphatase (µg/g)	Invertase (mg/g)
ICK	26 ± 2bc	3.12 ± 0.05b	0.32 + 0.022c	1.64 + 0.42b	7.61 + 1.92ab
IL	32 ± 4a	3.37 ± 0.10a	0.51 + 0.031a	3.56 + 1.10a	9.81 + 1.04a
FCK	24 ± 1c	3.09 ± 0.09b	0.27 + 0.027d	1.61 + 0.26b	7.12 + 0.56b
FL	27 ± 1b	3.28 ± 0.05a	0.39 + 0.020b	2.28 + 0.43b	8.6 + 1.19ab

Notes: Data are presented as mean values ± SD. Means followed by the different letter within the same column are significantly different (*p* < 0.05). ICK, IL, FCK, and FL are the intermittent treatment, lime combined with intermittent treatment, the flooding treatment, and lime combined with flooding treatment, respectively.
